# Over-the-Counter Supplement Overuse: A Rare Potential Cause of Arsenic Poisoning and Bradycardia

**DOI:** 10.7759/cureus.98745

**Published:** 2025-12-08

**Authors:** Jay Vankawala, Eden Altweiss, Alexandra M Glaeser

**Affiliations:** 1 Internal Medicine, Ronald Reagan University of California Los Angeles Medical Center, Los Angeles, USA; 2 Internal Medicine, University of California Los Angeles, Los Angeles, USA

**Keywords:** aresenic, bradycardia, heavy metal toxicology, herbal supplements, progressive cognitive decline

## Abstract

A 71-year-old woman with no significant past medical history presented with progressive cognitive decline, fatigue, and weight loss over two years. Her symptoms included bradycardia, anxiety, depression, and mild dyspnea on exertion. The patient followed a strict diet fortified with smoothies and an extensive regimen of 26 dietary supplements, consumed regularly over five years. Initial workup revealed sinus bradycardia at 40 beats per minute, mild cognitive impairment, and an elevated urine arsenic level (111.8 µg/L). After careful consideration of other possible etiologies, arsenic exposure was deemed a likely contributor to her symptoms.

Arsenic poisoning, typically caused by contaminated water or food sources, can present with fatigue, cognitive issues, and cardiovascular abnormalities such as bradycardia. Algae-based supplements, including spirulina and chlorella, are particularly susceptible to arsenic contamination due to their ability to absorb metals from the environment. In this case, the patient’s systemic symptoms were suggestive of arsenic toxicity, most likely secondary to dietary supplement use.

This case highlights the importance of health care providers obtaining a thorough dietary and supplement history. When risk factors are identified based on history, selective screening for heavy metal toxicity is warranted. Enhanced regulation of supplements and population-level education about their potential dangers are imperative to prevent adverse outcomes related to supplement use.

## Introduction

Arsenic is a metalloid element that is highly toxic and poses significant health risks [1]. Found naturally in the Earth's crust, arsenic can contaminate groundwater, industrial byproducts, and crops. Its toxicity arises from its capacity to interfere with fundamental cellular processes, such as inhibiting oxidative phosphorylation in mitochondria and disrupting the function of critical enzymes involved in energy production and detoxification [2]. Given its tasteless and odorless nature, arsenic exposure often goes undetected without specialized testing, complicating its identification and management.

Recent concerns have emerged regarding arsenic exposure through dietary supplements, particularly those derived from algae and herbal sources [3]. Supplements such as spirulina [4], chlorella [5], and ginkgo biloba [6] can accumulate high levels of arsenic when produced in contaminated environments. The inconsistent quality control and regulatory standards for dietary supplements heighten the risk of arsenic contamination. This may lead to significant health issues, such as cognitive impairment [7], peripheral neuropathy [8], increased risk of various cancers [9], and bradycardia [10] in consumers who are unaware of the potential risks associated with these products.

Historically, supplements were regulated as food in the United States. However, a change in classification in 1994 led to reduced oversight by the FDA [11]. The FDA no longer needs to ensure that nutritional supplements are safe and effective before they can be marketed. In part due to this deregulation, supplement use has increased substantially and was recently estimated to represent a $152 billion global industry [12]. According to the National Center for Health Statistics, which last surveyed supplement use among Americans in 2017-2018, 57.6% of adults aged 20 and older reported using supplements in the past month, a trend that was increasing across all age groups compared with prior years [13]. In a study of 121 supplements in Canada, high arsenic levels were found in 5% of products [14].

With the growing culture of dietary supplementation and the increasing reliance on these products for perceived health benefits, there is an urgent need to obtain a detailed dietary supplement history and consider the risk of arsenic exposure in the differential diagnosis of patients presenting with unexplained symptoms. Here, we present a case of profound bradycardia and cognitive dysfunction possibly due to arsenic toxicity in the setting of heavy supplement consumption.

## Case presentation

A 71-year-old woman with no significant past medical history presented to the hospital with chronic cognitive impairment, fatigue, weight loss, low energy, and anxiety, with a subacute worsening of symptoms over the past two years. The patient noted mild issues with memory recall approximately 15 years earlier, shortly before retiring as an elementary school teacher, which had not been concerning to her. Two years ago, she noticed a much more pronounced decline in her memory and word-finding abilities. She recalled one recent instance when she became lost while driving home from the supermarket. She denied any history of trauma or recent falls. The patient was the primary caregiver for her sister and husband, both of whom had significant illnesses. In addition to cognitive decline, she reported feeling fatigued, overwhelmed, anxious, and depressed. She had been seeing a physician approximately every two years but had been hesitant to fully disclose her condition.

Notably, for the past five years, the patient and her husband had followed a strict diet. They consumed only one meal per day, with all additional nutrients provided via smoothies or supplements. The reason for this dietary shift, according to the patient, was to improve overall health and prevent disease, though she had received no medical counseling to do so. The patient consumed 26 supplements daily (Table 1), with some taken multiple times per day. Over the last two years, she had unintentionally lost 30 pounds and reported worsening fatigue, decreased exercise tolerance, and mild dyspnea on exertion since the initiation of this restrictive diet coupled with supplements. Her constellation of lethargy, weight loss, diminished energy levels, and cognitive and mood changes prompted her to seek medical care.

**Table 1:** Supplements the patient was consuming, stratified by arsenic risk. Source: Reference [3].

Risk Level	Supplement Name
High Risk (greater likelihood of arsenic contamination due to environmental exposure or poorly regulated sourcing)	Spirulina (algae-based), Chlorella (algae-based)
Moderate Risk (moderate likelihood of contamination, often related to sourcing or the environment in which the supplement is grown)	Turmeric (herbal), Yerba Mate (herbal), Ginkgo biloba (herbal), Astragalus root (herbal), Olive leaf extract (herbal), Black seed oil (herbal), Bee pollen (natural product that may be influenced by environmental conditions)
Low Risk (low likelihood of arsenic contamination, typically due to synthetic production or rigorous purification processes)	Vitamin D3, Vitamin C, Omega-3 (fish oil), Zinc, Magnesium, Lutein, B-complex, Vitamin B12, Vitamin K2, Coenzyme Q10 (300 mg), D-ribose, Resveratrol extract, Acetyl-L-carnitine with folic acid, Coconut oil, Alive! multivitamin, Kimchi probiotics, Powdered greens (moringa, maca, cacao powder)

**Figure 1:**
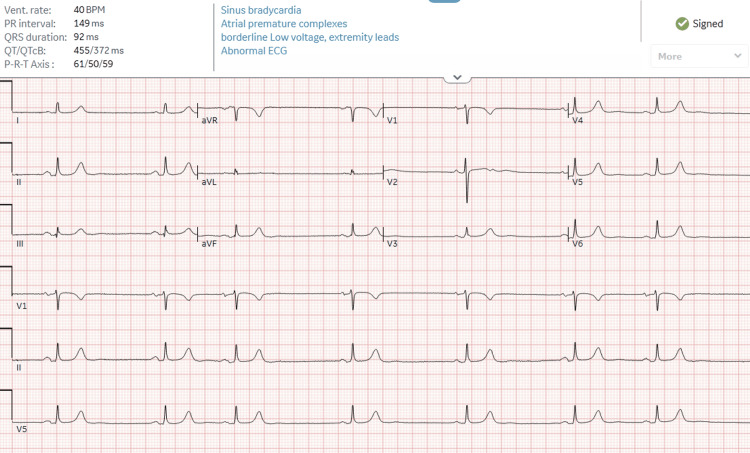
Admission EKG showing sinus bradycardia at 40 beats per minute with premature atrial contractions.

**Figure 2:**
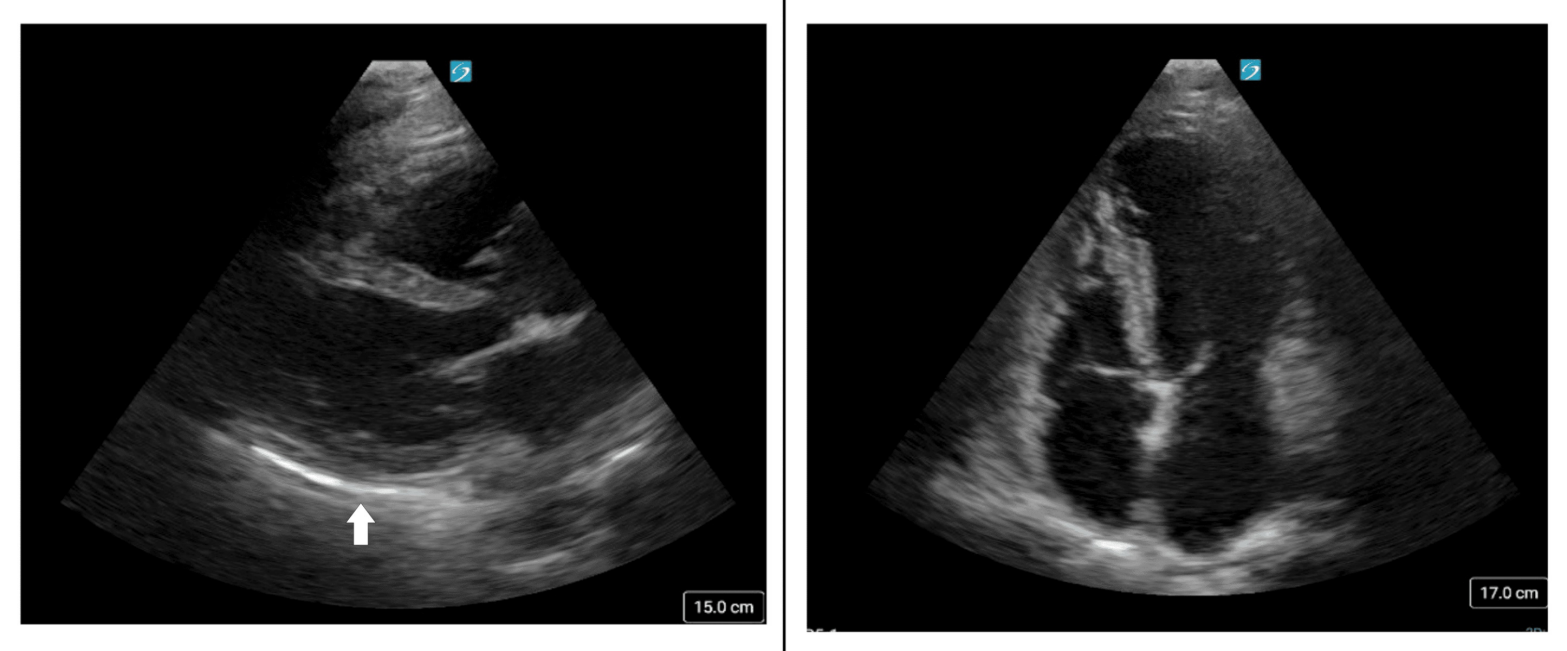
Cardiac point-of-care ultrasound showing the parasternal long-axis (left) and apical four-chamber (right) views. A trace pericardial effusion is seen (left, arrow). No wall-motion abnormality, valvular pathology, or reduction in ejection fraction was observed.

In the ED, the patient was found to have bradycardia, with a heart rate of 40 beats per minute. A chart review revealed that her bradycardia had been present since 2019, coinciding with the start of her restrictive diet. An EKG was performed, showing sinus bradycardia with premature atrial contractions, with no prior EKG available for review (Figure 1). The patient was unaware of this condition, and no previous workup or interventions had been performed. She was not taking any chronic medications and denied chest pain or significant shortness of breath at rest. Her systolic blood pressure was elevated to 176 mmHg on admission, and she denied a previous diagnosis of hypertension, although she did not check her blood pressure at home. Physical examination revealed an African American woman with a normal BMI, bradycardia without murmurs, and no appreciable rash. A limited point-of-care ultrasound revealed a normal estimated cardiac ejection fraction and trace pericardial effusion (Figure 2). Initial laboratory workup demonstrated normal electrolytes and thyroid-stimulating hormone (TSH) levels but leukopenia (Table 2).

**Table 2:** Pertinent laboratory results showing elevated arsenic levels with no other significant abnormalities.

Laboratory Value	Patient Result	Reference Range
Leukocyte count (cells/µL)	2,660	4,500-11,000
Sodium (mmol/L)	144	135-145
Potassium (mmol/L)	3.8	3.6-5.3
Creatinine (mg/dL)	1.14	0.6-1.3
Calcium (mg/dL)	9.3	8.6-10.4
Magnesium (mEq/L)	1.8	1.4-1.9
Phosphorus (mg/dL)	2.7	2.3-4.4
HIV	Nonreactive	Nonreactive
Rapid Plasma Reagin (RPR)	Nonreactive	Nonreactive
Thyroid-stimulating hormone (µIU/mL)	2.9	0.3-4.7
Vitamin B12 (pg/mL)	962	254-1,060
Vitamin B1 (nmol/L)	163	70-180
Vitamin D (ng/mL)	88	20-40
Urine arsenic (µg/L)	111.8	0-34.9
Urine arsenic-to-creatinine ratio (µg/g)	116.5	0-29.9

**Figure 3:**
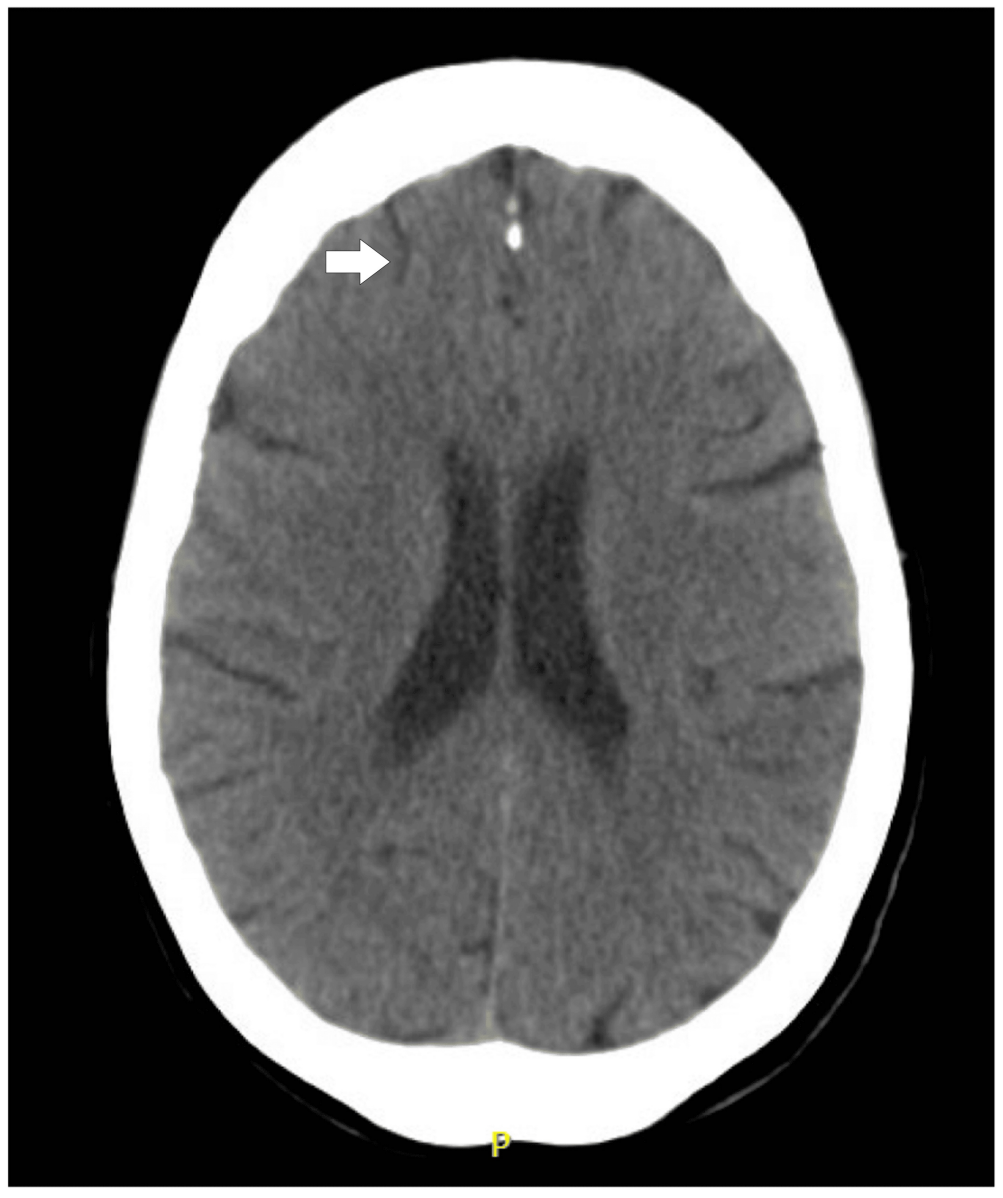
Non-contrast head CT showing normal cortical volume, preserved cortical architecture (arrow), and no evidence of an acute intracranial process.

Given her profound bradycardia, a cardiology consultation was obtained. The team recommended ambulation with telemetry to assess her heart rate response during activity. The patient showed appropriate heart rate variability, rising from around 40 to 80 beats per minute with ambulation, and she remained asymptomatic. Telemetry at night revealed sinus bradycardia around 35 beats per minute without pauses or heart block. It was determined that there was no clear indication for a pacemaker. A Montreal Cognitive Assessment (MoCA) was conducted for her cognitive concerns, with a score of 18 out of 30, consistent with mild-to-moderate cognitive impairment. Reversible causes of dementia, including nutritional deficiencies and infectious etiologies, were also ruled out (Table 2). A brain CT scan showed no acute intracranial process or notable cortical atrophy (Figure 3).

At this juncture, there was no confirmed diagnosis to explain her constellation of symptoms. Her bradycardia was possibly due to age-related sinus node degeneration, and her cognitive decline was suspected to represent early dementia or depression. Before anchoring on these diagnoses of exclusion, the medical team reconsidered her full clinical picture, cognitive, cardiac, and constitutional symptoms in the setting of high supplement intake and an unremarkable secondary workup. This prompted consideration of heavy metal toxicity, for which a 24-hour urine heavy metal screen was obtained. She was found to have an elevated urine arsenic level of 111.8 µg/L (reference range: 0-34.9 µg/L), with a urine arsenic-to-creatinine ratio of 116.5 µg/g creatinine (reference range: 0-29.9 µg/g creatinine, Table 2). The remainder of her urine heavy metal screening, including urinary lead and mercury levels, was normal. In consultation with toxicology, chelation therapy was not recommended.

Due to the patient’s appropriate cardiac response to ambulation and the absence of acute symptoms requiring hospitalization, she was discharged with recommendations to follow up with primary care, cardiology, and neurology, as well as to obtain an echocardiogram. Given the potential link between her symptoms and arsenic exposure, it was advised that she and her husband discontinue all dietary supplements. A nutritionist evaluated the patient and confirmed that her caloric intake was insufficient, providing her with a diet plan upon discharge. Additionally, it was recommended that her husband undergo heavy metal testing with his primary care physician. The patient’s supplements were never tested directly for heavy metals.

After discharge, the patient discontinued her supplements for a few weeks but subsequently resumed them at her husband’s insistence. At a 3-month phone check-in, she reported continued worsening of weight loss and cognitive function and is currently undergoing cardiac evaluation with her outside cardiologist. Given that she still consumes a spirulina smoothie daily, counseling was reinforced on the importance of discontinuing high-risk supplements, as they are likely contributing to her deteriorating health.

## Discussion

Arsenic poisoning occurs when an individual is exposed to high levels of arsenic, typically through contaminated water, food, or industrial sources. Common causes include drinking water contaminated with inorganic arsenic, especially in areas with naturally high arsenic levels, and consuming contaminated supplements, seafood, or produce. Symptoms of arsenic poisoning can vary but often include fatigue, cognitive decline, GI disturbances, hyperpigmentation, hyperkeratosis, and cardiovascular abnormalities such as bradycardia. Long-term exposure can increase the risk of cancer and neurological damage. Treatment of chronic arsenic poisoning primarily involves eliminating the source of exposure, such as discontinuing contaminated supplements or switching to clean water sources, along with supportive care [15]. The arsenic level at which symptoms of toxicity occur varies. Chelation therapy, guided by toxicologists, is largely reserved for acute arsenic exposure with severe symptoms and markedly elevated levels (>1,000 µg/L) [16].

Certain over-the-counter vitamins and supplements have been implicated in arsenic poisoning, primarily due to contamination from environmental sources. Algae-based supplements such as spirulina and chlorella are particularly susceptible to arsenic contamination, as these organisms absorb metals from their aquatic environments [17]. Several published studies have documented cases in which herbal remedies have led to symptomatic arsenic toxicity [18]. A study from Singapore investigated 74 individuals with chronic arsenic poisoning attributed to the use of traditional herbal remedies for asthma [19]. The poisoning had widespread effects on the skin, nervous system, and GI tract, and ten of these patients were later diagnosed with cancer. Other herbal supplements, including ginkgo biloba, yerba mate [20], and astragalus root [21], may also contain arsenic depending on the soil and water conditions where they are cultivated.

Arsenic toxicity can lead to bradycardia by disrupting several critical processes in the cardiovascular system [22]. It directly damages the myocardium, reducing the ability of cardiac cells to contract and conduct electrical signals effectively. Arsenic also interferes with mitochondrial enzymes essential for cellular respiration, thereby decreasing adenosine triphosphate (ATP) production, depriving heart cells of energy, and weakening their function. It can further cause electrolyte imbalances, particularly in potassium and calcium, which are vital for maintaining normal electrical conduction in the heart. Moreover, arsenic affects the autonomic nervous system by increasing parasympathetic activity and reducing sympathetic stimulation.

Arsenic toxicity was suspected to have contributed to this patient’s bradycardia, as reversible causes such as medication use, electrolyte disturbances, and hormonal imbalances were excluded through a normal workup. Her severe bradycardia, along with cognitive decline, chronic fatigue, and weight loss, suggested a systemic issue rather than a localized cardiac problem. Elevated urine arsenic levels, far above the normal range, strongly supported chronic arsenic exposure. In consultation with toxicology, it was determined that arsenic could be affecting the patient’s cognition and cardiac function. However, given the patient’s advanced age, we cannot be certain that it fully accounts for the cognitive decline and bradycardia. Other intrinsic causes of sinus node or autonomic dysfunction are possible, though arsenic toxicity remains a plausible explanation given its known effects on autonomic regulation. Another limitation of our report is the patient’s partial non-adherence to medical recommendations to discontinue supplements, which limited the ability to monitor symptoms and arsenic levels after cessation.

In the United States, dietary supplements, including natural herbs and botanical products, are regulated by the FDA under the Dietary Supplement Health and Education Act of 1994 [23]. However, these regulations are less stringent than those for prescription drugs. The FDA does not pre-approve supplements before they reach the market; instead, it relies on manufacturers to ensure product safety. Only after harm occurs may the agency investigate and remove supplements from the market. Although supplements are subject to Good Manufacturing Practices [24], contamination with heavy metals such as arsenic can still occur, particularly in products sourced from regions with poor environmental controls. In California, Proposition 65 [25] requires manufacturers to disclose when products contain harmful levels of certain chemicals, including arsenic, but compliance varies. As a result, consumers of natural herbs and supplements may still be at risk of arsenic toxicity, especially when products are not adequately tested for heavy metal contamination before being sold.

There is generally a lack of consumer awareness regarding the potential risks associated with supplement use. Often considered the “natural” way to optimize health, many consumers do not perceive these substances as medications and fail to report their use to health professionals, despite the fact that they can contain toxins, cause liver injury, or interact with anesthesia and other medications. Therefore, it is a health care provider’s duty to directly inquire about supplement use. From a public health perspective, it is imperative that there be more independent screening and regulation of supplements for safety before they reach consumers.

## Conclusions

Our patient’s reliance on multiple supplements and a restrictive diet raised concern for arsenic toxicity, with subsequent laboratory testing supporting this hypothesis. Although we cannot be certain that arsenic poisoning was wholly responsible for her symptoms, this case highlights the importance of a comprehensive and multidisciplinary approach when evaluating patients with nonspecific symptoms such as fatigue, weight loss, bradycardia, and cognitive decline. It underscores the need for a thorough medical history that includes detailed inquiries into dietary habits, supplement use, and environmental exposures. When risk factors are identified based on history, selective screening for heavy metal toxicity is warranted. Identifying chronic arsenic toxicity early is essential to prevent further health deterioration and ensure appropriate management. In addition to provider-level interventions aimed at obtaining a more complete history, this case emphasizes the need for systems-level change to better regulate supplements and provide accurate information to patients. Supplements can be beneficial to one’s health under the right conditions; however, it is vital to educate the general population about their potential harms, the importance of discussing supplement use with physicians, and how to discern which products are safest.
